# Disruptive innovation for inclusive renewable policy in sub-Saharan Africa: A social shaping of technology analysis of appliance uptake in Rwanda

**DOI:** 10.1016/j.renene.2020.12.091

**Published:** 2021-05

**Authors:** Olivia Muza, Ramit Debnath

**Affiliations:** a African Centre of Excellence in Energy for Sustainable Development, College of Sciences and Technology, University of Rwanda, Kigali, Rwanda; b Energy Policy Research Group, Judge Business School, University of Cambridge, Cambridge, United Kingdom; c Behaviour and Building Performance Group, Department of Architecture, University of Cambridge, Cambridge, United Kingdom; d International Energy Agency, Paris, France

**Keywords:** Energy transition, Off-grid system, Sub-saharan africa, Policy, Green growth, Disruptive innovation

## Abstract

Rural off-grid renewable energy solutions often fail due to uncertainties in household energy demand, insufficient community engagement, inappropriate financial models and policy inconsistency. Social shaping of technology (SST) of household appliances provides a critical lens of understanding the involved socio-technical drivers behind these constraints. This study employs an SST lens to investigate appliance uptake drivers in 14,580 households in Rwanda, such that these drivers can aid in policy design for green growth at the grassroots level. The methodology includes an epistemological review of non-income drivers of appliance uptake. Empirical analysis using a binary logistic regression, based on which disruptive innovation pathways were derived for fostering green growth. Results showed that appliance uptake was highly gendered and skewed across the Ubudehe (social welfare) categories. ICT-devices like mobile phones and radios had a higher likelihood of ownership than welfare appliances like refrigerator and laundry machines. Fans and cookers also demonstrated a greater probability of ownership. Disruptive innovation pathways were derived from leveraging the ICT-driven wave of appliance ownership, creation of service sectors through off-grid renewable solutions and promoting cleaner fuel-switching of cooking energy at the household level. Further policy implications were drawn to support the creation of consumption identities for green growth.

## Highlights

• Social shaping of technology analysis of appliance uptake in 14,580 household in Rwanda.
• Derived policy pathways using disruptive innovation for green growth at grassroots level.
• ICT driven solutions and cleaner cooking fuel switching are key for inclusive renewable policy.

## 1 Introduction

Gendered perceptions, preferences, ownership and benefits from electrical appliances for United Nations Sustainable Development Goals (SDG) 1 (poverty reduction), 7 (affordable and clean energy provisioning) and 17 (partnerships for the goals) remain an under-researched area in the off-grid and rural context of the Global South. Despite advances in technology, people in rural areas still use traditional stoves with biomass-based fuel for cooking and kerosene for lighting [1]. It has significant adverse health and well-being implications on the national burden of diseases and is extensively acknowledged in the literature [2]. It remains the case for 2.8 billion people globally [3]. In addition to reliance on traditional cookstoves, uptake of electrical appliances for household tasks, income generation and service-delivery continue to remain low. With the contemporary regime of micro-girds and renewable energy transition, it is crucial to understand the demand-side response of renewable technology innovations in resource-constrained setting (like rural areas) for designing good energy policy [4].

Higher uptake of electrical appliance is central to the achievement of SDGs and improved livelihood opportunities in poverty [5]. This study takes a two-step approach to understand the drivers of appliance uptake in African rural communities. First, a systematic literature review is conducted to identify factors critical in influencing appliance uptake in resource-constrained settings in the context of Global South and sub-Saharan Africa. Second, the performance of local communities in appliance uptake is investigated in rural Rwandan using binary logistic regression on Integrated Household Living Conditions (EICV5) micro dataset. In doing so, this study seeks to understand the process of technology diffusion in rural Africa and establish vital policy indicators for the socially inclusive energy transition. Social Shaping of Technology (SST) framework is used to visualise and integrate the social inclusiveness, community and gendered appliance uptake [6]. SST explores how the design and implementation of technology are patterned by a range of ‘social’ and ‘economic’ factors as well as narrowly ‘technical’ considerations [7]. This approach aided us in understanding the complex relationships between technology innovation diffusion of renewables in rural Rwanda and the social factors influencing the process of diffusion. The key indicators of SST are derived through an in-depth epistemological review of appliance ownership, gender dynamics, technology change and socio-cultural identities.

Globally, technological innovation is happening at a fast pace, and the knowledge of its diffusion at a local level is vital to understand the process of ‘just’ energy transition and green growth. At a provincial level, energy transitions are complicated because, despite new technological innovations and solutions, traditional appliances continue to co-exist with electrical appliances, so there has been the use of multiple energy sources. A significant corpus of literature on energy transition in Africa had been focussing on the dualities of energy use and storage in rural and low-income communities through the lens of ‘energy stacking’ [1,8]. Green growth literature shows that such technology and energy stacking behaviour is influenced by collective identities of a community that in turn, shape the consumption characteristics [9]. However, there is a significant literature-gap in examining the implications of the renewable energy transition and energy stacking behaviour, especially on the appliance uptake drivers.

The literature on the acceptance of renewable energy technologies had been concentrated on exploring the micro-grid technologies with the assumption that when such technologies are accepted, electrical appliances are also automatically accepted [10]. This assumption influences the policy mechanisms to treat electrical appliances as a secondary component of renewable energy allocations, that has a snowballing effect on the distributional energy injustice of appliance ownership and renewable technology diffusion in rural and low-income communities in the Global South [11,12].

Our assumption is that we can minimize the snowballing of distributive injustices by enabling disruptive innovation and green growth at the grassroots level. Therefore, we investigated the social shaping of technologies in Rwanda and derive pathways for social inclusivity in technology diffusion and higher appliance uptake using the theory of disruptive innovation (after [13]. The application of the theory of disruptive innovation in a bottom-up manner is also called Disruption from Below [14,15].

The primary research question of this study is, *how does specific appliance uptake get shaped by the social-technical drivers in a resource-constrained setting?* To address this question, the following objectives are formed:

- To understand the drivers of household appliance ownership in rural Rwanda within the theoretical scope of SST.
- To examine the gendered influence on appliance uptake in Rwanda and establish vital indicators of the socially inclusive energy transition.
- To derive higher appliance uptake pathways using the lens of disruptive innovation to support green growth in low-income communities.

**Fig. 1:**
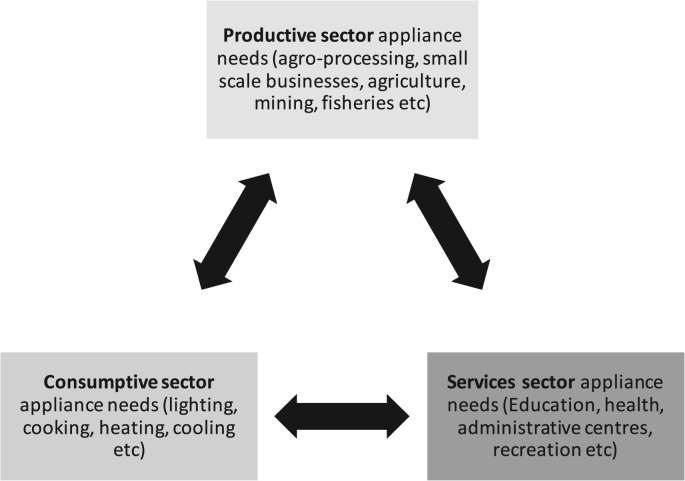
A conceptual local-level disruption from below approach for sustainable energy transition in rural areas.

Addressing these objectives is not only crucial in answering the research question, but it also forwards a transformative local level appliance uptake strategy through disruptions from below, which is consumptive-productive-service oriented. It also embedded the collective intelligence needed for removing the informal barriers to green growth at the grassroots level [9]. This derived approach outlines the novelty of this study, as a gendered and socio-technical narrative of consumptive-productive-service oriented appliance uptake would critically aid in designing appropriate policies for sustaining small and medium-sized rural enterprises, equitable allocation of appliance needs in resource-constrained setting (like in education, healthcare, administrative centres, entertainment and recreation), and for enabling green growth at a household level through appropriate energy services (lighting, cooking, heating, cooling, etcetera). The consumptive-productive-service oriented appliance uptake approach for the green energy transition in rural and resource-constrained areas of Global South is illustrated in Fig. 1.

The paper is structured as follows: section 1 defined the scope and conceptual framework of this study. Section 2 illustrates an in-depth literature review of appliance ownership drivers in low-income communities of Global South. This section also accentuates the need for a gendered perspective in designing good energy policies at the community and grassroots level. Additionally, this section also connects the critical links between non-income drivers and SST, and community influence on energy transition through disruptive innovation and green growth perspective. Section 3 explains the overall methodology of this study and the use of EICV5 dataset of Rwanda for quantitative analysis using a binary logistic regression. Section 4 illustrates the results and links the critical implications of the results with the broader goals of the renewable energy transition. The discussions are presented in section 5. Finally, conclusion and policy implications are presented in Section 6.

## 2 Literature review

### 2.1 Gendered implications of appliance uptake in sub-Saharan Africa and the Global South

Women’s involvement in decision-making in domestic energy remains an under-researched area, especially in low-income communities. Understanding the importance of gender roles in energy consumption is crucial for sustainable energy policymaking at a household and neighbourhood scale, especially for rural mini-grid planning [16,17]. However, much of the present studies on gender and energy use is derived from empirical findings of the Global North. Few studies that lay the foundation of gendered influence on energy use and well-being in low-income communities show a strong relationship between the quality of the built environment, use of space and appliance ownership [8,18,19], but do not report any critical variables that can help in the prediction of energy demand in resource-constrained settings. In rural areas, this uncertainty associated with energy demand forecasting remains one of the critical barriers of mini-grid proliferation which adds financial risk to the investors [16].

Mini-grids and solar home systems (SHS) are crucial tools of off-grid electrification in remote rural and island communities. It has significant sustainable development and poverty alleviation implications in areas like sub-Saharan Africa, Asia and the Latin American countries. Despite the successes recorded in many countries of the Global South, the sub-Saharan African mini-grid electrification narrative portrays failure and limited success [20]. The significant constraints against off-grid renewable energy-based electrification programs include lack of technical and managerial knowledge needed to run and maintain the systems, low-energy demand density, uncertainty of energy demand from households, disperse homestead, insufficient community engagement, inappropriate financial models, policy inconsistency and lack of political will [[20], [21], [22], [23], [24], [25], [26]]. These challenges limit the socio-economical developmental impacts of the rural off-grid renewable energy transition programs.

However, electrification alone cannot solve all development problems, but access to electricity acts as a gateway to other forms of development assistance (D. F [27]. A recent study by Dhanaraj, Mahambare, & Munjal, (2018) in urban India have shown that improving household access to welfare appliances like refrigerator and washing machine to women living in low-income improves household welfare. The access to such welfare appliances improves the convenience of women that frees up their time from doing subsistence-based household chores (like cooking, cleaning and washing). This time is usually used in income-generating activities that contribute critically to the improvement of household welfare in low-income communities [28]. It illustrates the importance of gendered capacity building in access to modern appliance uptake.

Additionally, in a broader sub-Saharan African study [20], have recommended that a gendered capacity building and technology transfer can substantially solve diffusion problems of renewable micro-grids in rural areas. It can aid in better access to modern appliances in a rural household. It, in turn, can foster ways for better financial models through electrification-gender-entrepreneurship nexus at local-level.

Gendered appliance uptake perspective at the local level is vital to establish the envisaged consumptive-productive-service link (see Fig. 1). To establish this link, it is imperative to understand the gender-related choices and constraints of appliance uptake from a Social Shaping of Technology (SST) perspective (as mentioned in section 1). Literature shows that SST and gender-related factors are influential in determining appliance ownership in rural areas, as traditional appliances still co-exist with modern appliances even as the small-scale entrepreneurship in sub-Sharan Africa has increased [29,30]. SST implicates in a triangulated manner such that socio-political acceptance, community acceptance and market acceptance remain in synchronisation [30]. In a similar context in India, Angelou & Bhatia [31], have reported that in both rural and urban households social processes and household structures determine appliance uptake, rather than sole income-related drivers as claimed by energy ladder concepts. Such drivers are called ‘non-income’ drivers of appliance ownership that are critical players in reshaping the demand of a particular appliance or technology [28,30,32,33]. Understanding these non-income drivers and the mechanisms of SST in low-income is essential for sustainable renewable micro-grid planning in rural areas as household moving out of poverty become the first purchaser of electrical appliances [1,20,34].

### 2.2 Social shaping of technology in appliance uptake in the Global South

A recent growing body of literature exclusively focuses on the non-income drivers of appliance uptake in conjunction with the social shaping of technology (SST) theories. These studies are mostly focussed on the Global South and poverty alleviation context, as the consumption behaviour in these areas is highly complex, socio-culturally layered and have distinct rural-urban characteristics on technology choices [20]; D. F. [27]; D [1,8,28,32,[35], [36], [37], [38], [39], [40]]. The common thread between these studies is towards understanding the relationship between technology and social life in low-income and resource-constrained setting. As mentioned in section 2.1, a better understanding of technology and society can help in better off-grid renewable planning, execution and delivery, and is a must for realising UN SDG – 7. In this purview, we synthesise information on technology innovation and its influence on resource-constrained and rural societies of the Global South.

Wu, (2008) used an ethnographic approach to understand the complex relations between technology and social life in a Chinese rural setting and to explore the logic and dynamics of integrating new technology products into their everyday life. The author found that for quick technology adaptation in a rural setting, appropriation of technology is vital across the socio-cultural layers of rural areas [41]. also commented that for good energy policymaking, it is essential to understand everyday habitus and the gendered views on technology vis-à-vis household appliances. In a similar note, Bisaga & Parikh, (2018) have used a practice-based approach in examining the technology adaptation of solar home systems (SHS) in rural Rwanda. They found that social practice changes dramatically that, in turn, influences the social shaping of SHS. Due to this complex non-linear SST process, the energy consumption in a rural household does not increase linearly with time or with more appliance. Frederiks, Stenner, & Hobman [42], further expanded on the technology innovation and SST viewpoint in appliance uptake across economic classes to derive policy action points on more cost-effective and mass-scalable behavioural solutions to encourage renewable and sustainable energy use among consumers. In their in-depth review, the authors found that many studies reported that the consumers benefit from technological innovation in their daily practices, without which their well-being is affected (similar arguments made in Roberts, Hope, & Skelton, (2017)). Although these studies are from the Global North, the technology-well-being interdependencies remain valid in the Global South context as well [43]. Our study further expands the understanding of such interdependencies by assessing socio-technical drivers of appliance uptake in Rwanda, which can assist renewable-based microgrid providers to identify ways of improving innovations suitable for off-grid consumers.

**Fig. 2:**
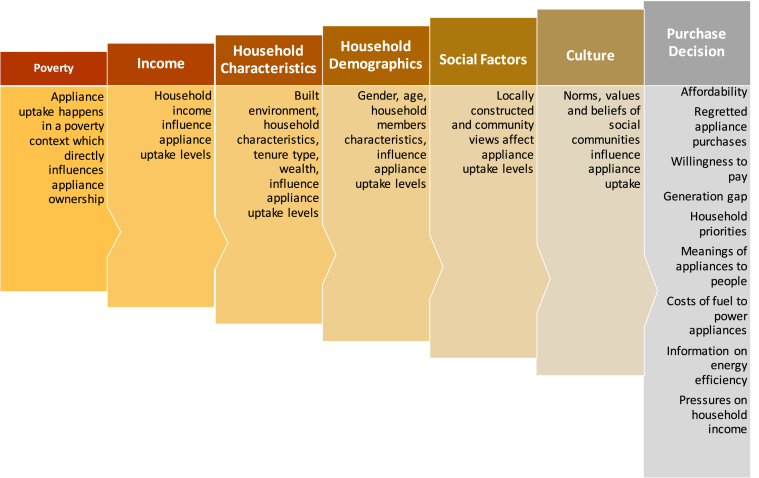
Authors synthesis of the factors influencing social shaping of appliance uptake in low-income communities based on current literature.

A more in-depth literature search exhibited studies that have analysed the SST drivers of appliance uptake from an epistemological viewpoint. These studies are presented in Table 1, and we synthesised a flowchart of such SST weighed drivers of appliance uptake in poverty using the information presented in Table 1. The synthesised flow diagram is illustrated in Fig. 2.

**Table 1:** Epistemology of the influence of technology innovation and SST on appliance uptake in low-income and rural context (source: Authors).

References	Epistemological arguments in relation to SST and technological innovation of appliance uptake	Methodology
[44]	Study found that 53% of adults reported regretting purchasing an electrical device at some point, and 23% regretted making such a purchase within the past year. The regretted consumption is triggered by the pace *of technology change* making the device obsolete. *[Note: this study is not from the Global South, but the implications are important for this study]*	National sample survey (n = 2000) across socio-economic classes, personal interviews and social practice theory of regretted consumption
[28]	Change of household practices and built environment leading to shifting of energy intensive practices from outdoors to indoors in urban poverty of Mumbai. The respondents were *coming out of poverty*, technology change did not concern them, but rather they were *first-time buyers* of ‘modern’ appliance on a ‘subsistence’ basis.	Questionnaire survey of 1224 slum rehabilitation housing occupants using social practice theory. Analytical technique involved co-variance based structural equation modelling.
[32]	Examined patterns of ownership of televisions, refrigerators and washing machines as welfare appliances. Authors found a hierarchy of preference in appliance uptake owing to physical quality of built environment and demographic characteristics. Race (color) and religion was also found to a crucial *social force* shaping appliance ownership. Apart from this affordability, expenditure share and automobile ownership were also critical drivers.	Publicly available nationally representative household survey data from Brazil, India and South Africa. Analytical technique involved logit modelling and boosted regression tree.
[45]	*Changing social practice* shape consumer behaviour towards adoption of solar home systems (SHS) in rural Rwanda. SHS acts as a technological force behind changing perception towards new technology adoption and energy stacking dynamics.	Empirical enquiry using social practice theory of 265 respondents.
[46]	Social shaping of technology like air conditioning (AC) is important for addressing cooling needs in warming Global South. Solutions should be beyond improving AC efficiency and *focus on passive buildings and city design, innovative cooling technologies and parsimonious use of ACs.* Technology diffusion and innovative solutions are key to future cooling needs in the Global South.	Variable degree day (VDD) method on a global grid.
[47]	Importance of *local markets*, order of successive appliance purchases and corresponding income levels. Other parameters influencing appliance uptake are climate, degree of urbanisation, electrification rate.	Country specific interviews on appliance ownership and use patterns of appliances; Dataset from World Bank (Living Standards Measurement Study); national census datasets of Brazil, Mexico, Nicaragua, Panama, Peru and South Africa.
[48]	Social factors (family, roles & status, age & life cycle stage); physical factors (occupation and economic status) and marketing mix (promotion and placement) were key drivers of *social shaping of consumer behaviour* towards appliance uptake.	Questionnaire survey of 200 households in Iraq, and structural equation modelling.
[49]	*More female participation* and energy stacking dominate small and medium enterprises of street food service (SFS) in Senegal, South Africa and Rwanda. The need for affordable and accessible modern energy services in SFS shape the technology diffusion in these areas.	Mixed-method interview of 751 respondents.
[19]	Gender-sensitive built environment design influences energy practices associated with appliance uptake in low-income settings.	Systems analysis and interview of female occupants in slum rehabilitation housing of Mumbai, India
[16]	Improving energy use surveys to improve accuracy of energy prediction in micro-grids for rural areas. It can aid in better technology diffusion and appliance uptake.	Surveyed and measured in eight mini-grids.
[18]	The benefits of energy services and new technology *are not equally distributed* between men and women in rural energy transition due to *socio-cultural practices and norms*. It affects the *energy culture* that, in turn, determines the success of off-grid electrification program.	Qualitative examination of Mpanta solar mini grid in rural northern Zambia using energy culture perspective.
[50]	Electricity service quality determines the willingness-to-pay for grid-connected electricity bills (i.e., appliance use) in rural India. Indian policymakers can increase electricity prices in exchange for *improved services and better technology.*	ACCESS survey across 715 villages in six Indian states

### 2.3 Disruption innovation for socially inclusive energy transition and green growth

Disruptive innovations do not attempt to bring better products; instead, they disrupt and redefine the existing market trajectory by introducing products and services that are more convenient, more straightforward and less expensive [51]. Disruptive innovation plays a vital role in energy transition theory as it emerges from constructivist sociology and evolutionary economics [[52], [53], [54]]. Transition theorist often presupposes that disruptiveness is a requirement for system innovation, however, among modern thinkers, transition theory has been more attendant to broader societal structures and institutions [55,56]. In energy system-based transition, innovations have a distinctive emphasis on the hierarchy that creates barriers for sustainable innovations and transitions [57]. [20] demonstrated such barriers in micro-grid transition in rural Africa and reported that technological transition and innovation must be complementary to the renewable transition to enable higher uptake of appliances. As mentioned in section 2.1, the lack of such complementary planning of technological innovation and appliance uptake often results in the long-term failure of off-grid rural electrification systems due to system lock-in Ref. [25].

Moreover, it is the uncertainty in the electricity load prediction among rural consumers that reduce the performance of off-grid systems. The complex causes of this uncertainty are illustrated in Table 1 and Fig. 2, that cluster deeply around the social shaping dimensions of technology. Winskel [58], say that the disruption of the energy system is itself a necessary and welcome enabler of the shift to more sustainable and more rapidly decarbonised energy systems.

Building on [56]’s argument, we envisage that disruptive innovation in rural renewable energy transition (especially in sub-Saharan Africa) would mean replacing traditional energy sources and appliance with the modern form of electrical appliances. It would need replacement of traditional cooking stoves and charcoal irons through a socially inclusive electrical appliance uptake strategy which should be consumptive-productive-service sector-oriented (see Fig. 1). To this effect, we use Christensen’s theory of disruptive innovation [13] and examines four questions; ‘*Are renewable energy providers in the market improving along a trajectory of sustaining innovation? Have renewable energy providers overshot customer needs? Do renewable energy providers possess the capability to respond to disruptive threats? Are energy providers floundering as a result of innovation?’* By answering these questions, we can identify three critical elements of disruptions, first, rate of improvement of appliance uptake. Second, we identify the distinctively different trajectory to improvements of the uptake of appliances in the study area. Third, is the critical element of understanding the pathways of sustaining innovations in a specific socio-economic setting.

The challenges associated with socially inclusive energy transition in rural areas are those related to practicalities of implementation, location in remote areas with steep terrain and impoverished customers which affects sustainability, limited local technical and managerial skills, low energy demand, inadequate availability of supply components, and unproven financing models [20,59]. Public acceptance, social acceptance and local acceptance of renewable micro-grids are crucial for inclusive transition [60,61].^1^ Community co-ownership (COO) has been widely discussed in the literature as a disruptive strategy of local acceptance of renewable micro-grids [[62], [63], [64]].

Disruptive innovation-led technological change is considered as a powerful tool of systems change for green growth [14]. Christensen’s approach of disruptive innovation is also referred to as ‘Disruptions from Below’ that is critical to bottom-up approaches of system change. Nogami & Veloso, (2017) further expanded on it and derived four key action points, namely, sustaining innovation, overshooting consumer needs, response to consumer threats and floundering as a result of innovation. This study builds on this theoretical lens of disruption from below.

Solar PV is an example of ‘Disruptions from Below’, where the technology was initially expensive, and currently, it is one of the cheapest forms of energy [14,65]. The uptake of solar was initially driven by a subsidy that resulted in low production costs due to mass-market adoption. With the increase in the quality of the technology, the demand grew, and the prices declined. It began to disrupt the mainstream market of household solar energy systems. Similar inferences can be drawn for personal computers, digital cameras and mobile phones [66].

Disruption from Below is also crucial for the clean energy transition in the Global South as it provides a space for technology transfer and collaboration in the South-South trade [67]. In a recent study on renewable energy policies and transition in 34 African countries, authors reported that countries such as Egypt, Ethiopia, Mauritius, Namibia, Rwanda, South Africa and Uganda have direct, integrative and enabling energy policies that address questions of recognitional and distributive energy justice [68]. Thus, providing Rwanda with an edge for establishing green growth paradigm at household-level through disruptive innovation-induced appliance uptake. It will, in turn, ensure that the future social shaping of technology will inherently be cleaner and greener.

A recent systematic literature review by Capasso, Hansen, Heiberg, Klitkou, & Steen [9], have shown that a significant driver to long-term green growth is consumption habits and behavioural change. It transverses across individual consumption decisions to collective identities shaping consumption clusters that, in turn, shape the trajectory of businesses and innovations. Smulders, Toman, & Withagen [69], inferred that while the role of technological progress is vital for green growth, but it does not necessarily lead to green growth. There is a need for directing technological progress towards greener technologies. This study explores the trajectories of appliance uptake in rural Rwanda through the lens of social shaping of technology. It establishes the trajectories of green growth at a household-level through disruptive innovation as per the conceptual framework illustrated in Fig. 1.

## 3 Data and method

### 3.1 Data

**Fig. 3:**
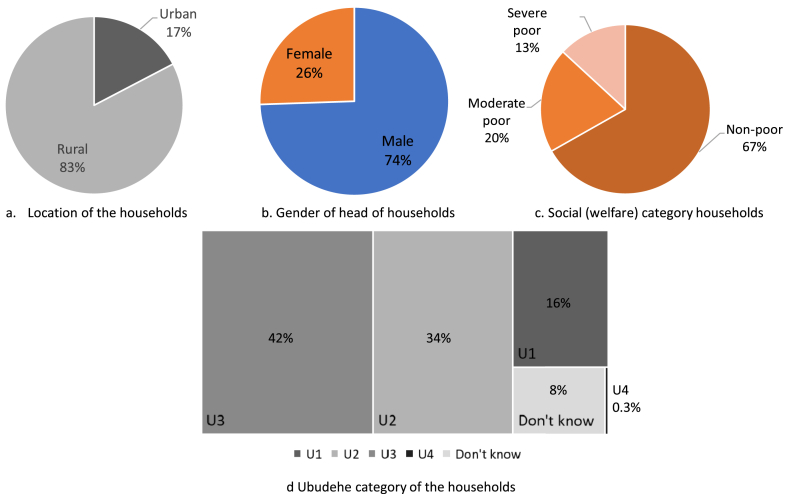
Demographic characteristic of the households under study (n = 14,580).

This study is based on Rwanda Integrated Household Living Survey (EICV) dataset for the year 2010/11 (EICV3), 2013/14 (EICV4) and 2016/17 (EICV5). The EICV5 dataset interviewed 14,580 households, representing 64,314 people [70]. The EICV5 survey shows that 38.2% of the population was poor in 2016/17, as compared to 39.1% as measured by the EICV4 survey of 2013/14. During the same period, extreme poverty went from 16.3% to 16.0% [70]. The EICV5 report also states that the reduction in poverty between EICV5 and EICV4, respectively, was not statistically significant. The poverty gap rate, which measures the gap between people’s spending and the poverty line, also showed a non-significant change to 11.7 in 2016/17, from 12.0 in 2013/14 [70]. The summary of inequality and poverty rate for 2010–2017 is shown in Table 2. The population of Rwanda is 12.63 million (as per 2019) with more than 70% of the population living in rural areas [71]. The demographic characteristics of the households analysed in this study as per the EICV5 dataset are illustrated in Fig. 3.

**Table 2:** Summary of inequality and poverty rates in Rwanda (2010–2017).

	EICV3: 2010/11	EICV4: 2013/14	EICV5: 2016/17
Gini coefficient	0.49	0.44	0.42
Headcount poverty rate	44.9∗	39.1∗	38.2
Poverty gap rate	14.8∗	12.0∗	11.7
Sample size	14,308∗	14,419∗	14,580

(Source: NISR, EICV3, EICV4, EICV5. Note: ∗includes panel sample).

In 2007, the Government of Rwanda (GoR) launched its Economic Development and Poverty Reduction Strategy (EDPRS) of which Vision 2020, is part of its strategy to address the three pillars of sustainable growth for jobs and exports, Vision 2020 *Umurenge* Program (VUP) and good economic governance. The VUP is an Integrated Local Development Program to ‘Accelerate Poverty Eradication, Rural Growth, and Social Protection’. It uses the existing decentralisation system and leverages technical and financial assistance to accelerate the rate of poverty reduction in Rwanda. The aim is to eradicate extreme poverty by 2020 [72]. The VUP is organised around three components, first intends to revive public works through community-based approaches. Following components innovate with credit packages to tackle extreme poverty as to foster entrepreneurship and off-farm employment; and the third components includes direct support to improve access to social services and basic amenities [72]. This study aims to contribute to the VUP strategies by forwarding socially inclusive energy transition pathways. Additionally, the gendered perspective employed in this study aligns with VUP’s strategy of economic growth enabler by off-grid electrification of small and medium enterprises in rural areas [72].

**Fig. 4:**
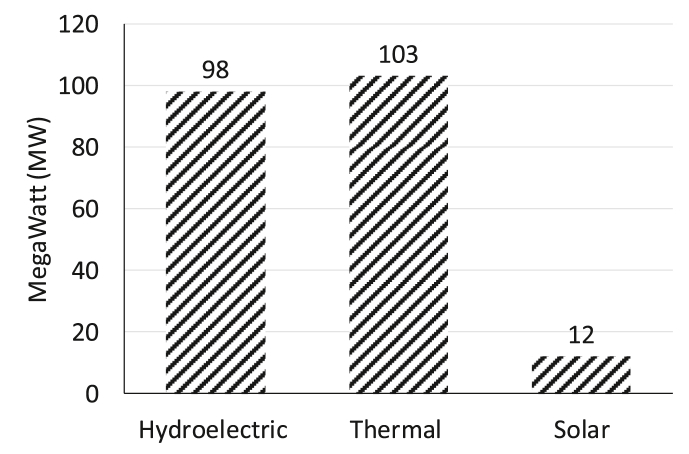
Installed capacity in Rwanda, 2018 (Source [73]).

The GoR envisions 100% electricity access by 2024, with 52% on-gird and 48% off-grid electricity generation. It currently has 218 MW (MW) of installed generation capacity, and its national electrification rate is estimated to at 30% (12% in rural areas, 72% in urban areas) [73]. The present installed capacity is illustrated in Fig. 4; there are 1.7 million households without power in 2018. The current challenges in electrification include misalignment of power supply and demand, limited financing for off-grid companies and limited affordability of electricity solutions for rural households and businesses [73]. Through this study, we intend to create higher off-grid appliance uptake pathways for socially inclusive energy transition (as mentioned in section 1).

### 3.2 Empirical analysis

A binary logistic regression model is used to examine the drivers of appliance ownership and investigate the influence of location of household, the gender of the head of household (HoH), population consumption quintiles, social (welfare categories) and the *Ubudehe* category. In Rwanda, social class is ranked using the Ubudehe welfare ranking. Ubedehe is a traditional community-driven collective action of solving problems. It is Rwanda’s best known indigenous solution to poverty alleviation [74]. The Ubudehe categorisation is crucial to the success of the VUP program for efficient resource allocation and direct credit transfer mechanisms. The Ubudehe categorisation is illustrated in Table 3, where, it is logical to imply that lower categories (1 & 2) would own a smaller number of household appliances than the higher categories (3 & 4). Besides, based on the GoR’s definition and categorisation of the Ubudehe categories, we assume that category 1 are in *extreme poverty* who cannot afford even the basic electrical appliances, and exclude it from the regression model. Similarly, social (welfare) category classifies the population-based on poverty line RWF 159,375 (∼USD 168) per year. Population living above it are categorised as ‘Non-poor’, where population below RWF 105,064 (∼USD 110) per year are identified as ‘Extremely/Severely poor’ (see Table 4).

**Table 3:** Ubudehe categories as per the Government of Rwanda (source [75]]).

Ubudehe category	Characterisation
Category 1	Very poor and vulnerable citizens who are homeless and unable to feed themselves without assistance.
Category 2	Citizens who can afford some form of rented or low class owned accommodation, but who are not gainfully employed and can only afford to eat once or twice a day.
Category 3	Citizens who are gainfully employed or are even employers of labour. Within this category are small farmers who have moved beyond subsistence farming, or owners of small and medium scale enterprises.
Category 4	Citizens classified under this category are chief executive officers of big businesses, employees who have full-time employment with organizations, industries or companies, government employees, owners of lockdown shops or markets and owners of commercial transport or trucks

**Table 4:** Variable list and their description.

Dependent variable	Data type (Binary: 1 = Yes, 0 = No)	
Appliance type	[1] Radio with or without CD player; [2] Mobile telephone; [3] TV set; [4] Satellite dish; [5] Video/DVD player; [6] Decoder; [7] Music system; [8] Computer and accessories; [9] Cooker; [10] Laundry machine;	[11] Electric fan; [12] Sewing machine; [13] Refrigerator/freezer; [14] Electric generator; [15] Electric hotplate; [16] Power stabilizer; [17] Still camera; [18] Video camera; [19] Printer; [20] Water filter
**Independent variable**	**Data type (Discrete)**	**Descriptive**
Location of household	Urban [REF]; Rural	[Note: Since the scope of this study is rural-centric. We use the urban variable as a reference [REF] category in the analysis]
Ubudehe category	Category 1 (U1); Category 2 (U2); Category 3 (U3); Category 4 (U4) [REF]	Category 4 (see Table 3) is used as a reference category.
Gender of the head of household (HoH)	Male [REF]; Female	[Note: We report only the ‘female’ gender related results. Male is the reference category]
Quintiles	Q1: poor Q2 Q3: middle Q45: rich [REF]	Consumption quintiles as per EICV5 classification [70]. The Q4 and Q5 (Q45) is the reference category, classified as rich households.
Social (Welfare) categories	[1] Severely poor [2] Moderately poor [3] Non poor [REF]	Poverty classification as per the [70] EICV5 dataset. The poverty line is drawn at RWF 159,375 (∼USD 168) per year and the extreme poverty line at RWF 105,064 (∼USD 110) per year. [1 USD = 947.25 RWF; Dec 2019 rate

The primary dependent variable of appliance ownership in the EICV5 dataset is examined through the question *‘How many durables does your household own?’* It enlists 29 electrical and non-electrical durables, (of which 20 were electrical appliances), with electrical appliance ownership treated as a binary variable (1 = Yes, 0 = No). The variable list is illustrated in Table 4. See appendix for the descriptive statistics of the variables and its correlogram.

In this study, under the binary logistic model, the estimated value of the dependent variable (Appliance = 1; Non-appliance = 0) is interpreted as the probability that the technology diffusion of an appliance in a household (HH) is driven by the independent explanatory variables (as per Table 4). The empirical model is represented as (see eq. (1)),

(1) $$Y_{i}=b_{0}+b_{1}Location_{i}+b_{2}Gender_{i}+b_{3}Social_{i}+b_{4}Quintiles_{i}+b_{5}Ubudehe_{i}+u_{i}$$

$$\left\{ \begin{array}{l} y_{i}=1\;if\;a\;particular\;appliance\;is\;present\;\\ y_{i}=0\;if\;the\;particular\;appliance\;is\;not\;present\; \end{array}\right.$$

where, $Y_{i}$ is a binary variable indicating whether the specific appliance is owned by the HH (Yes/No); Jun, Kim, Jeong, & Chang [76], also performed a similar contingent valuation methodology employing dichotomous variables. A binary Dummy variable (1 = Yes, 0 = No) for each of the appliances (see Table 4) was created to fit the definition of $Y_{i}$. The aim is to determine how each appliance has penetrated within the social context of the independent variables, and the likelihood of its diffusion based on the location, gender, social category and Ubudehe category (as illustrated in eq. (1)). Epistemological evidence from the literature show that non-income drivers (like the independent variables of eq. (1)) promote the higher likelihood of technology diffusion (appliance uptake) in poverty that is critical in designing social inclusive energy transition policies (see Table 1 and Fig. 1). $Location_{i}$ is also a binary dummy variable (1 = Yes, 0 = No) accounting for ‘rural’ location. $Gender_{i}$ is a dichotomous variable that explains the gender of the head of household (HoH), with Male = 1 and Female = 0. Binary dummy variables for social categorisations ($Social_{i}$) accounted for each of the welfare categories (see Table 4) as 1 = Yes, 0 = No. Similarly, the five quintiles ($Quintiles_{i})$ are accounted for as binary variables (1 = Yes, 0 = No) by creating dummy variables for each of the quintiles (Q1, Q2 …, Q5). Referring to the [70] EICV5 classification, we merged the high-income consumption quantile Q4 and Q5 as Q45 to improve the interpretability of the results. $Ubudehe_{i}$ represented four categories (see Table 4), and dummy variables were assigned to create binary values for each of the categories (1 = Yes, 0 = No); $u_{i}$ is the error term. Therefore, the modified equation is illustrated in eq (2).,

(2) $$\;Y_{i}=b_{0}+b_{1}urban_{i\left[REF\right]}+b_{2}rural_{i}+b_{3}male_{i\left[REF\right]}+b_{4}female_{i}+b_{5}nonpoor_{i\left[REF\right]}+b_{6}moderatepoor_{i\left[REF\right]}+b_{7}severepoor_{i}+b_{8}Q1_{i}+b_{9}Q2_{i}+b_{10}Q3_{i}+b_{11}Q45_{i\left[REF\right]}+b_{12}U1_{i}+b_{13}U2_{i}+b_{14}U3_{i}+b_{15}U4_{i\left[REF\right]}+u_{i}$$

$$\left\{ \begin{array}{l} y_{i}=1\;if\;a\;particular\;appliance\;is\;present\;\\ y_{i}=0\;if\;the\;particular\;appliance\;is\;not\;present\; \end{array}\right.$$

## 4 Results

**Fig. 5:**
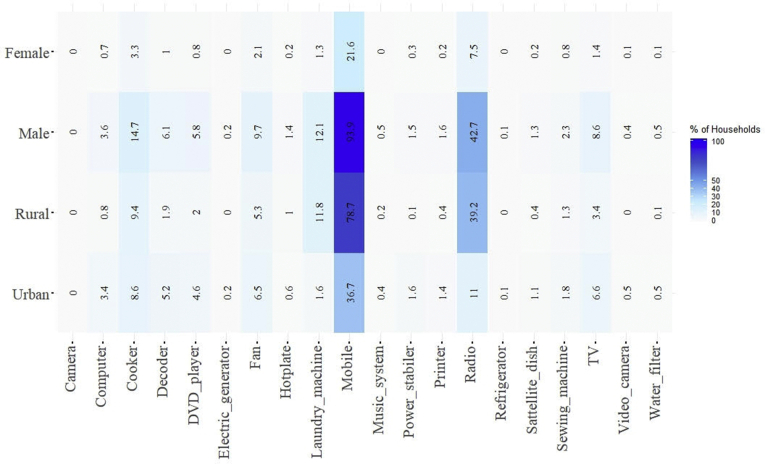
A heatmap illustrating gendered appliance ownership in urban and rural Rwanda (n = 14, 580).

The EICV5 micro dataset that surveyed 14,580 households recorded appliance ownership as household durables (as reported in section 3.2, Table 4), Fig. 5 shows the distribution of gendered appliance ownership as in urban and rural Rwanda. It can be observed that information and communication technologies (ICT) devices like the radios and mobile phones have the most appliance uptake across the rural (78.7%) and urban (36.7%) household with 93.9% of the mobile phones are owned by the male head of households (HoH), female-headed household showed 21.6% of the total mobile phones ownership. Welfare appliance like the refrigerator and washing machine uptake is low across the rural-urban boundaries of Rwanda. It can be seen from Fig. 5 that rural households had 11.8% of laundry machine (washing machine) uptake where the urban area has a 1.6%. The refrigerator (including freezers) uptake is 0.10% urban and 0.08% rural, with more male representation in the appliance uptake. Appliances like TV sets and fans had higher uptake in the urban areas (6.6% and 6.5%, respectively) with a strong male-centralism (8.6% and 9.7%, respectively). Cooker shows higher appliance uptake in the rural area; however, it is a combination of both electrical and non-electrical variants. Also, hyper-modern and skill-generating appliance uptake like computers were high in urban areas (3.4%), and, male-centric (3.6%). Computer uptake by the female is 0.7% of the surveyed sample. Fig. 5 distinctively indicates the gendered appliance uptake pattern in Rwanda.

[Note: Male and Female are referred to the gender of the head of household (HoH); of which 75% were males and 25% were female, according to ECIV5 survey demographic characteristics [70]].

**Fig. 6:**
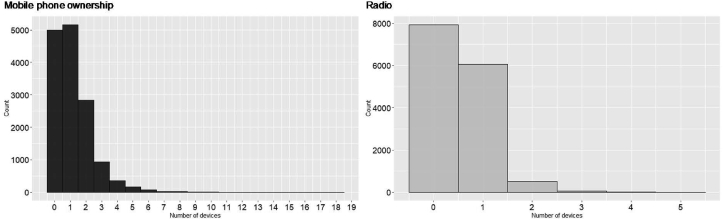
Household ICT device ownerships in Rwanda (n = 14,580).

Further breakdown of dominant ICT device uptake is illustrated in Fig. 6 that shows that most of the households had at least two mobile phones. However, radio ownership is at a maximum of 1 radio per household, most of the households have no radios, even though it has a higher ownership frequency (see Fig. 5). Mobile phone driven ICT diffusion across socio-economic layers have been reported to have distinctive social and sustainable development impacts, especially for women and low-income communities [77,78]. From a social shaping of technology perspective (SST), mobile phones (ICT) diffusion have helped micro-entrepreneurs in rural Rwandan communities to expand their business by developing new business and social networks [79]. The higher penetration of mobile phones, as illustrated by the EICV5 dataset (see Fig. 6), shows better prospect for the realizability of the VUP (Vision 2020 *Umurenge* Program) program to foster Rwanda’s sustainable development goals.

**Fig. 7:**
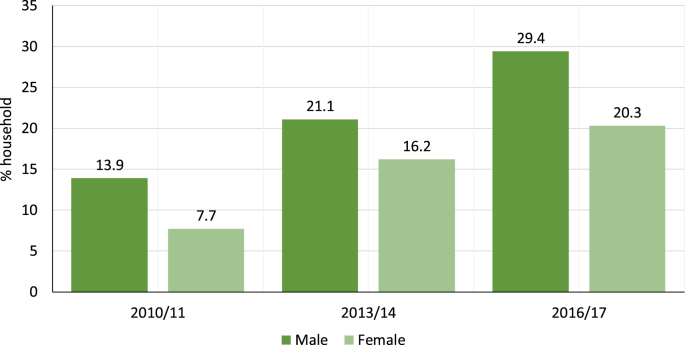
Household with electricity as the main source of lighting by the HoH gender (Source: EICV3 (n = 14,308), EICV4 (n = 14,419) and EICV5 (n = 14,580) dataset).

**Fig. 8:**
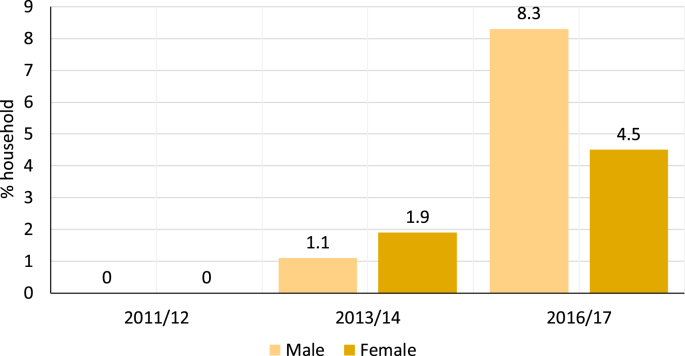
Household with solar panel as the main source of lighting by the HoH gender (Source: EICV3 (n = 14,308), EICV4 (n = 14,419) and EICV5 (n = 14,580) dataset).

Apart from ICT devices, the electrification rate is also a key indicator of development, especially concerning the progress in UN SDG – 7. A descriptive panel data representation of EICV 3, EICV 4 and EICV 5 dataset show that overall share of electric lighting (bulbs, tube lights, LEDs, etcetera) has increased between 2010 and 2017 (see Fig. 7). More importantly, the diffusion of solar-lighting devices illustrated the progress in off-grid electrification in Rwanda (see Fig. 8). The overall uptake of solar-based lighting is increasing, with 0% share in 2010/11 to approximately 13% in 2016/17; of which 8.3% was owned by male HoH and 4.5% by female HoH (see Fig. 8). Higher influx of off-grid solutions like solar home lighting systems shows the further propensity of appliance uptake (from the SST perspective) in Rwanda that can help the government to realise its poverty alleviation and the national VUP targets.

**Fig. 9:**
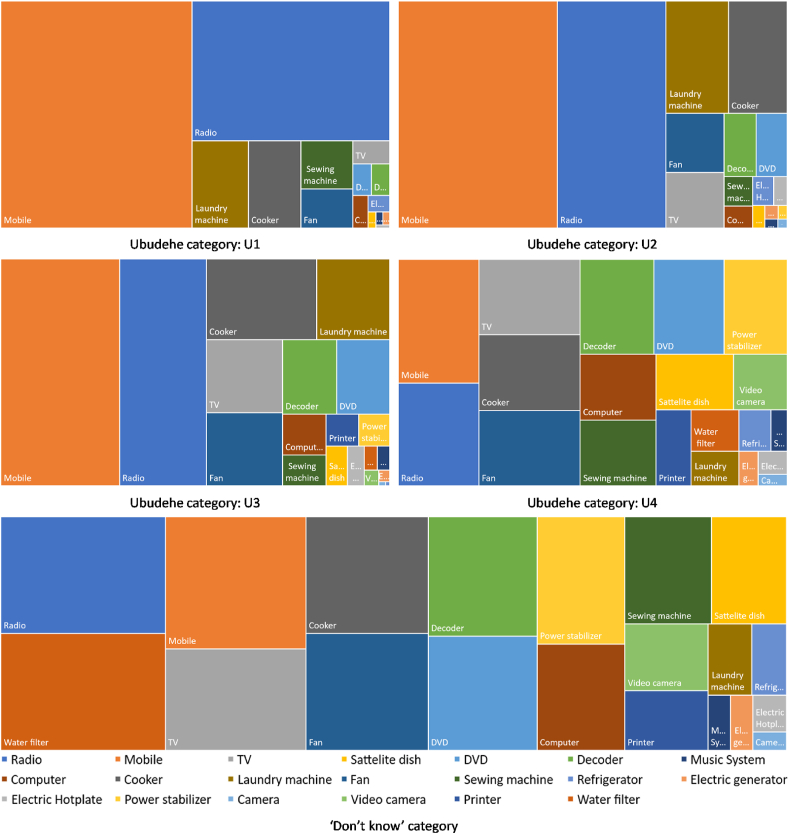
Appliance uptake as per the Ubudehe categories in EICV5 dataset (n = 14,580) [note: ‘Don’t know’ category is a mixed category where the respondents did not know their Ubudehe category, as per the EICV5 datasheet [70]].

The appliance uptake among the welfare categories of Rwanda (Ubudehe, see Table 3) shows a distinct distribution of appliances across it (see Fig. 9). The appliance uptake in U1 and U2 show higher ownership of radio and mobile phones; however, categorically the diffusion of fans, laundry machines and TVs are higher in the U2 category. Further segmentation of the appliance ownership is distinct in the U3 and U4 of the Ubudehe categorisation (see Fig. 9). It can be interpreted as the middle-class (and higher) way of consumption. The upper socio-economic consumption pattern is evident in the ‘*Don’t know’* category, where there is a characteristic mix of hyper-modern appliances that improve the ‘convenience’ factor of daily life, vis-à-vis higher household welfare. This argument is based on Sovacool’s interpretation of energy service ladder across the socio-economic segment (see Table 5 of [80]).

**Table 5:** Estimated binary logistic regressions of appliance uptake in rural Rwanda (yes: 1, no: 0).

Variables		Radio	Mobile phone	TV	Satellite dish	DVD player	Decoder	Music System	Computer	Cooker (Electric and non-electric)	Laundry Machine
Location	Rural									**−1.798∗**	
										(0.166)	
	Urban [REF]		**2.334∗∗**								
			(10.319)								
Social (welfare) category	Non_poor [REF]	**0.592∗∗**								**1.292∗∗**	
		(1.807)								(3.638)	
	Moderate poor	**0.350∗∗**	**0.303∗∗**							**0.779∗**	
		(1.419)	(1.354)							(2.178)	
	Severe Poor										

Quintiles	Q1	**−1.127∗∗∗**	**−1.405∗∗∗**	**−3.560∗∗∗**		**−3.288∗∗**				**−1.789∗∗∗**	**−1.252∗∗∗**
		(0.324)	(0.245)	(0.028)		(0.037)				(0.167)	(0.286)
	Q2	**−0.886∗∗∗**	**−1.239∗∗∗**	**−2.854∗∗∗**		**−2.212∗∗∗**	**−3.403∗∗**			**−1.761∗∗∗**	**−0.765∗∗∗**
		(0.412)	(0.290)	(0.058))		(0.110)	(0.033)			(0.172)	(0.465)
	Q3	**−0.775∗∗∗**	**−0.935∗∗∗**	**−2.447∗∗∗**	−**3.106∗∗∗**	**−2.513∗∗∗**	**−2.807∗∗∗**	**−2.040∗∗∗**	**−3.347∗∗∗**	**−1.684∗∗∗**	**−0.467∗∗∗**
		(0.461)	(0.392)	(0.087)	(0.045)	(0.081)	(0.060)	(0.130)	(0.035)	(0.186)	(0.627)
	Q45 [REF]	**−0.455∗∗∗**	**−0.607∗∗∗**	**−1.317∗∗∗**	**−1.475∗∗∗**	**−1.279∗∗∗**	**−1.364∗∗∗**	**−0.930∗**	**−2.140∗∗∗**	**−0.938∗∗∗**	
		(0.635)	(0.545)	(0.268)	(0.229)	(0.278)	(0.256)	(0.394)	(0.118)	(0.392)	
Ubudehe category	U1	**−0.184∗**		**−1.117∗∗∗**		**−0.852∗∗**	**−0.933∗∗**		**−1.066∗∗**		
		(1.202)		(0.327)		(0.427)	(0.393)		(0.344)		
	U2	**0.627∗∗∗**	**0.428∗∗∗**	**0.438∗∗**		**0.459∗∗**	**0.422∗∗**		**−0.829∗∗∗**	**0.502∗∗∗**	**0.616∗∗∗**
		(1.871)	(1.534)	(1.550)		(1.582)	(1.524)		(0.436)	(1.652)	(1.851)
	U3	**0.933∗∗∗**	**0.847∗∗∗**	**1.113∗∗∗**	**0.498∗**	**1.085∗∗∗**	**1.160∗∗∗**			**1.125∗∗∗**	**0.819∗∗∗**
		(2.541)	(2.323)	(3.044)	(1.646)	(2.961)	(13.795)			(3.079)	(2.269)
	U4 [REF]	**1.779∗∗∗**		**2.554∗∗∗**	**2.621∗∗∗**	**2.550∗∗∗**	**2.624∗∗∗**		**1.590∗∗∗**	**2.041∗∗∗**	**1.038∗**
		(5.927)		(12.862)	(13.748)	(12.813)	(1.881)		(4.904)	(7.699)	(2.825)
Gender	Female										
										
	Male [REF]	**0.983∗∗∗**	**0.624∗∗∗**	**0.757∗∗∗**	**0.626∗∗**	**0.860∗∗∗**	**0.632∗∗∗**	**0.954∗**	**0.330∗**	**0.361∗∗∗**	**1.184∗∗∗**
		(2.672)	(1.867)	(2.133)	(1.870)	(2.364)	(1.881)	(2.595)	(1.391)	(1.434)	(3.267)
Model-fit summary	Constant	−1.985	−1.063	−21.885	−21.762	−21.979	−21.139	−21.113	−21.498	−1.418	−3.031
	Nagelkerke R Square	0.174	0.220	0.448	0.292	0.396	0.447	0.180	0.384	0.346	0.122
	Hosmer and Lemeshow Test (Chi-square)		**66.453∗∗∗**								**16.465∗**

∗∗∗, ∗∗ and ∗ represent levels of significance at 1%, 5% and 10%, respectively. Odds-ratio are presented in parentheses. Reference cases are denoted as [REF].

As discussed in section 2, lower-income (and some middle-income) households in Global South portray a dynamic energy stacking behaviour that creates a mix of traditional and modern appliance uptake shapes the uptake of a specific technology. Interestingly, this study shows that mobile phones and radio (both ICT-devices) penetrated across the socio-economic sections of Rwanda (see Figs. 6 and 9) that creates a platform for ICT-driven sustainable development policies for meeting the targets of VUP. Donner, (2006) have reported that an increase in mobile phone ownership among the rural areas expanded microentrepreneurial network for grassroots businesses. Future off-grid planning in Rwanda must account for this ICT-diffusion, especially to reduce the gendered disparity in its ownership (as illustrated in Fig. 5), and to foster ICT-driven sustainable development. Better access to ICT-devices, especially for women, would empower them and help them build a more resilient rural-business network, which is crucial for disruptive innovation in resource-constrained and low-income communities [15].

**Fig. 10:**
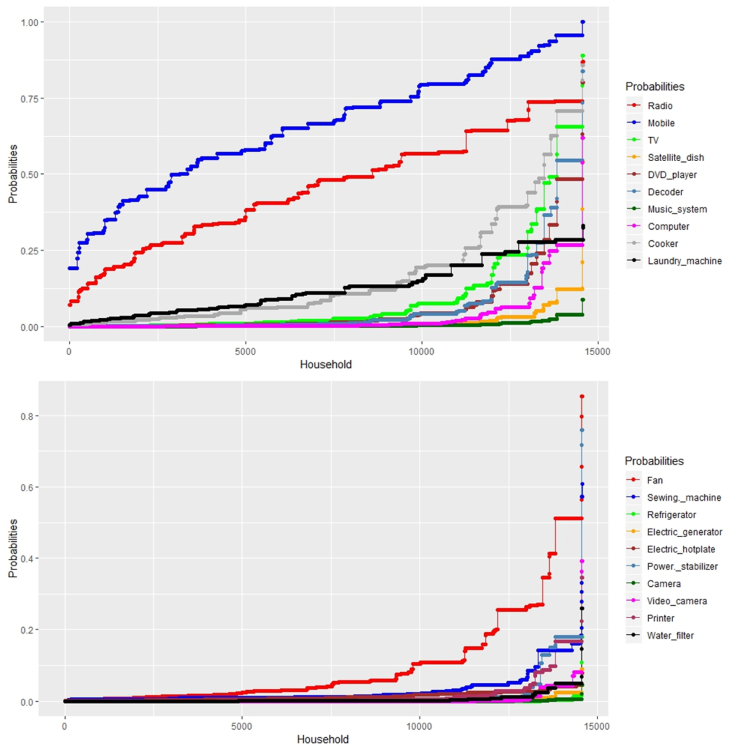
Predicted probabilities of appliance uptake in Rwanda (n = 14,580).

The binary logit regression results are presented in Table 5, and Table 6 illustrates the interdependencies between appliance uptake and its drivers. The socio-economic and gendered drivers considered (see Table 4) in this study were drawn from epistemological evidence from the social shaping of technology (SST) and its effect on appliance uptake (see Table 1 and Fig. 2). Appliance-wise uptake analysis shows that devices like mobile phones, radio, TV, cooker (electric and non-electric) and fan have a higher probability of uptake (see Fig. 10). In general, the appliance uptake is highly gendered and location-specific in Rwanda, which is a critical clue for SST. Based on the reference cases [REF], it can be seen from Table 5, Table 6 that ‘urban’ location and ‘male’ dominate the appliance ownership across the spectrum of socio-economic variables under study (see eq (2)). Moreover, the cluster of significant coefficients of appliance ownership can be found in higher-income Ubudehe (U3 and U4) and higher-quintiles (Q45), see Table 5, Table 6.

**Table 6:** Estimated binary logistic regressions of appliance uptake in rural Rwanda (yes: 1, no: 0) (continued from Table 5).

Variables		Fan	Sewing Machine	Refrigerator	Electric generator	Electric Hotplate	Power stabilizer	Camera	Video camera	Printer	Water filter
Location	Rural					**−0.578∗∗∗**					
						(0.786)					
	Urban [REF]										
										
Social (welfare) category	Non_poor [REF]										
										
	Moderate poor										
										
	Severe Poor										

Quintiles	Q1	−**2.420∗∗∗**	**−2.240∗**			**−2.443∗**					
		(0.089)	(0.106)			(0.087)					
	Q2	**−2.164∗∗∗**	**−2.482∗**								
		(0.115)	(0.084)								
	Q3	**−1.793∗∗∗**	**−1.414∗∗∗**			**−0.800∗∗∗**				**−2.697∗∗∗**	**−2.501∗∗**
		(0.166)	(0.243)			(0.449)				(0.067)	(0.082)
	Q45 [REF]	**−1.035∗∗∗**	**−1.653∗∗∗**				**−2.466∗∗∗**		**−2.700∗**	**−1.659∗∗∗**	**−1.814∗∗∗**
		(0.355)	(0.191)				(0.085)		(0.067)	(0.190)	(0.163)
Ubudehe category	U1	**−0.431∗**									
		(0.662)									
	U2	**0.367∗∗**	**−0.584∗∗**						**−1.885∗∗**		
		(1.443)	(0.558)						(0.152)		
	U3	**1.047∗∗∗**		**−1.537∗**		**0.876∗**				**0.744∗∗**	
		(2.850)		(0.215)		(2.400)				(2.104)	
	U4 [REF]	**2.766∗∗∗**	**2.094∗∗∗**	**2.560∗∗∗**	**2.528∗**		**3.053∗∗∗**		**2.001∗∗∗**	**1.714∗∗∗**	**2.191∗∗∗**
		(15.888)	(8.089)	(12.930)	(12.535)		(21.181)		(7.399)	(5.551)	(8.944)
Gender	Female										
										
	Male [REF]	**0.395∗∗∗**				**0.569∗**				**0.610∗∗**	
		(1.484)				(1.767)				(1.841)	
Model-fit summary	Constant	−21.548	−21.085	−21.730	−21.905	−21.711	−21.394		−21.18	−21.749	−21.852
	Nagelkerke R Square	0.306	0.175	0.433	0.291	0.074	0.445		0.401	0.326	0.258
	Hosmer and Lemeshow Test (Chi-square)		**13.411∗**						**16.523∗**		

∗∗∗, ∗∗ and ∗ represent levels of significance at 1%, 5% and 10%, respectively. Odds-ratio are presented in parentheses. Reference cases are denoted as [REF].

Information and communication technology (ICT) devices have higher appliance ownership across the consumption quintiles, income groups and the Ubudehe categories (see Table 5). The positive sign of the regression coefficient between radio ownership and social(welfare) category shows that this device has a higher probability of ownership among the non-poor (Odds Ratio (OR) = 1.807) and moderately poor (OR = 1.419) categories. The predicted probabilities across the households are illustrated in Fig. 10. Besides, the likelihood of radio uptake by a male is higher belonging to higher income Ubudehe categories (U3 and U4). However, it decreases across the U1 category (see Table 5). Similarly, mobile phone ownership has a higher likelihood of uptake in urban areas (OR = 10.319) and among the male members (OR = 1.867). A positive relationship is also amongst the higher income Ubudehe categories, while the negative signs across the social (welfare) categories show that mobile-phone ownership may be independent of the relative welfare status.

While ICT devices are modern critical indicators of SST (also mentioned in section 2), the demand of energy service for comfort, convenience and cleanliness (3Cs, after [81]) is also a critical indicator of distributive justice, especially in low-income housing [82]. The convenience devices/appliances save time that has welfare effects [80]. Convenience appliances uptake like TV, Refrigerator, Laundry machine, Computer, Electric hotplates and sewing machine shows a significant skewness towards male-dominance and social-economic hierarchical categories (see Table 5, Table 6). TV has the highest likelihood of uptake in the Ubudehe categories U3 (OR = 3.044) and U4 (OR = 12.862), respectively (see Table 5). Whereas the U1 category, has a negative correlation with the TV uptake (see Table 5), indicating socio-economic barriers. A similar negative correlation paradigm can be observed between TV uptake and the social welfare categories, which indicate there may be other SST-forces influencing its uptake.

Laundry machine and refrigerators are critical welfare appliances in low-income households that reduce the drudgery of women by saving time [83]. In Rwanda, the likelihood of uptake of the laundry machine is higher among the higher income Ubudehe categories, U2 (OR = 1.851), U3 (OR = 2.269) and U4 (OR = 2.852) with a significant male dominance (OR = 3.267) (see Table 5). The highest likelihood of refrigerator ownership is among the U4 category (OR = 12.930) showing high income-based inequality in welfare appliance uptake in Rwanda (see Table 6). Similarly, the likelihood of uptake electric hotplate decreases significantly in rural areas (OR = 0.786) and lower-income households. It is highly likely that electric hotplate will be present in a male-headed household belonging to U3 (and above) categories (see Table 6). The dominance of higher-income U3 and U4 categories in the total appliance ownership is also evident from Fig. 9.

Interestingly, the ownership of cookers (both electric and non-electric) have a significant influence on location and social categories (see Table 5). The EICV5 dataset does not specify the type of cooker; however, our analysis shows that the rural location has a significantly negative effect on its ownership (OR = 0.166). It can be due to the widespread use of firewood for cooking in rural and low-income households. Such fuel stacking behaviour is extensively discussed in the current literature. The use of firewood for cooking contributes to high indoor air pollution in rural Rwanda that has significant health burden, especially on women and children [84]. Furthermore, a higher likelihood exists among the moderately poor (OR = 2.178) and non-poor (OR = 3.368) households (see Table 5). Similar likelihood trend applies for the U2, U3 and U4 categories, respectively indicating that the SST behind cooking appliance ownership is highly income dependent. It has crucial policy implications for clean cookstove initiatives.

The energy service for comfort was primarily availed through fans, with a negligible representation of energy-intensive equipment like air conditions (ACs) in the EICV5 dataset. The ownership and uptake of the fan also has a high skewness towards the higher-income households (see Table 6). However, ownership of fans has a higher predicted probability, indicating its greater diffusion rate among the Rwandan households (see Fig. 10). A critical appliance in Rwanda is the high ownership of power stabilisers in a higher-income household that can imply on the low power quality in the country. The U4 (highest income) households have the highest likelihood of ownership of power stabilisers (OR = 21.181) as they have the most significant share of household appliances (see Fig. 9). Thus, low-income households may also refrain from buying appliances due to power quality issues, such that unstable voltage and frequent load-shedding may damage the appliances. The repair of damaged appliances further causes an economic burden, that may lead to a poverty trap in many households. Similar, causality between repair of appliances and poverty was also observed in low-income households in Mumbai, India [28]. These are the major SST forces of appliance uptake in Rwanda which have a significant location, higher-income and gendered influence. These forces will further shape the appliance uptake trajectory, as illustrated in Fig. 10.

ICT-devices like mobile phones have the fastest uptake trajectory and shapes the appliance diffusion of its associated technologies (see Fig. 10). shape ICT-driven energy governance and social development policies, deeper penetration of mobile phones can provide a better and more robust ecosystem for mobile-based solar home system solutions. Success stories of such initiatives can be seen from Ghana, South Africa, Nigeria and Kenya; critical lessons can be learnt from Smith, [85]. Moreover, ICT-provides a robust micro-entrepreneurial platform for local business to grow and expand, successful business models are illustrated by Donner, [79]. The rise and success of M-Pesa in Kenya [86] as a mobile phone-based financial bank also shows the importance of efficient utilisation of ICT-platforms in sub-Saharan Africa. The ICT-driven M-Pesa’s pay-*as*-you-go model is being utilised by disruptive solar companies (like M-Kopa Solar) to provide rent-to-own energy products. M-Kopa Solar is bringing in a low-cost, off-grid and socially inclusive energy revolution in Kenya [87]. Such disruptive innovation (or Disruption from Below) is needed in Rwanda to create socially inclusive renewable energy transition and green growth in rural and resource-constrained areas. The synthesis of pathways for disruption from below is derived in the next section.

## 5 Discussion

This study investigated the social shaping of technology of appliance uptake in Rwanda and devised a green growth approach at the grassroots level for inclusive renewable technology transition in rural and low-income communities. The green growth implications were forwarded through the lens of disruptive innovation at the grassroots, i.e., Disruptions from Below. The empirical modelling consisted of binary logistic regression on a national-level household appliance ownership and socio-economic dataset (see equation (2) in section 4 for regression function).

Results showed that the highest probability of appliance uptake in rural areas lies with information and communication technology (ICT) devices like mobile phones and radios (see Figs. 9 and 10). The next highest likelihood of ownership lies with devices like fans and cookers (see Fig. 10). The highest probability of ICT device ownership illustrates the impending information technology revolution in these areas. Green growth in this context would mean taping into this collective identity associated with ICT technology diffusion in the rural and low-income areas that will shape the consumption clusters. A similar argument for green growth at a policy level was also forwarded by Ref. [14]. Disruption from below for Rwanda in ICT would mean supporting micro-enterprises in rural areas to build on the growing ICT-segment through subscription-based services. For renewable energy transition, it would mean development of a subscription model for solar energy systems at a household or community level and at the same time promoting higher appliance uptake through attractive financial schemes.

The uptake of welfare appliances like refrigerator and laundry machine has a higher likelihood of adoption by middle-income (U3 Ubudehe category) and upper-middle-income (U4 Ubudehe category) households, as compared to lower social classes (see Table 5, Table 6 and Fig. 9). Enabling welfare appliance uptake in low and middle-income households is critical as it promotes household welfare and well-being by empowering women from freeing-up their time from daily household chores [28,83]. An inclusive renewable energy transition must account for the infrastructural support for such appliances that can support green growth by enabling comfort and convenience through the demand for modern energy services. R [82]. have shown that enabling access to modern energy services for comfort and convenience in low-income settlements of India and Brazil can have significant distributive energy justice effects. Disruptive innovation (i.e. disruption from below) in case of welfare appliance for rural Rwanda would mean reducing purchase cost and operating cost. Purchase costs can be reduced to attractive financial schemes or subscription-based models along with renewable energy solutions. Moreover, operational costs can be reduced through improved energy efficiency, improvement of power quality and reliability; which will, in turn, increase the inclusivity of off-grid renewable power system. Lessons in this regard can be learned from the last-mile business model of SELCO-India [88,89].

TV ownership was also observed to have a higher likelihood of uptake among the U2, U3 and U4 category (see Table 5 and Fig. 9). It further adds to the policy implications towards improving the welfare of women as [90] had shown that higher TV exposure improved rural Indian women’s status. Similarly, Fig. 10 shows that the cooker has a higher probability of uptake in rural households. Although the dataset used in this study did not differentiate between the source of cooking energy (see Table 4), energy stacking behaviour is prevalent across the Ubudehe categories. Switching the cooking energy mix with a cleaner fuel mix should also be a critical objective of inclusive renewable energy transition. This finding also supports Rwanda’s national biomass energy strategy [91]. [68] have stated that enabling cleaner fuel mix is critical for energy justice and green growth in African countries. Lessons of cleaning the fuel mix at the household level using off-grid renewable solutions can be learnt from Ref. [92].

Synthesis of disruption from below pathways for consumptive-productive-service oriented appliance uptake approach as per the conceptual framework (see Fig. 1) of this study is presented in Table 7.

**Table 7:** Disruption for Below pathways for green growth and inclusive renewable energy transition in for appliance uptake in Rwanda.

Synthesising disruption innovation for bottom of pyramid as per applied Christensen’s Theory	Consumptive-productive-service sector appliance needs based on social shaping of technology in rural areas	Implications for green growth and inclusive renewable energy transition in rural and resource-constrained areas
**Sustaining innovation**	• Improving the diffusion of appliances-based on the appliance pattern as shown in Fig. 10 • Creating local micro-entrepreneurship driven financial models. Roadmap is in place in the Vision 2020 Umurenge Program (VUP) of the government of Rwanda. • Skill development and community-led energy management initiatives with off-grid solutions in rural households.	• Consumption clusters for green growth should be created around the ICT devices as it is the fastest diffusing technology in Rwanda (see Fig. 10). • Encouraging and supporting frugal innovation with service-based business model for enabling greater utility derivation from mobile phones. • Fuel switching of cooking energy in rural households with cleaner modes like solar or biomass is critical for green growth in Rwanda.
**Overshooting consumer needs**	• Anticipating the uncertainties associated with household energy demand due to diffusion of electrical appliances will improve the resilience of off-grid renewable energy systems. It will in turn promote more appliance uptake across socio-economic classes. • Financial models and incentives for greater uptake of welfare appliances in the rural areas. It should be in-line	• Enabling infrastructure for welfare appliance uptake and ICT devices with off-grid electrification planning to avoid consumer overshooting. • Better and more equitable tariff plan as per the Ubudehe categories can promote consumption-centric green growth for inclusive renewable energy transitions. The ongoing VUP program can be an ad-hoc platform for such energy policy instruments.
**Response to consumer threats**	• Better understanding of behavioural routines and collective identities through energy use and social shaping of technology surveys. • The government should leverage the VUP platform to experiment with behavioural public policymaking. Recent example of such data-driven policymaking approach from public narratives and public policy discourses can be found in Ref. [82].	• The growing ICT ecosystem in Rwanda should be leveraged with just energy policymaking at the grassroots level. Micro-entrepreneurship at rural level to support the current wave of appliance uptake in Fig. 10 can aid in agile responses to consumer threats. • Special schemes for the U2 and U4 Ubudehe category can promote equity in appliance uptake. Therefore, fostering green growth at the bottom of the pyramid.
**Floundering as a result of innovation**	Increase system efficiency, reliability, provision of super-efficient appliances, reduce consumer tariffs and offer services that will improve consumer willingness to pay. Better repair and maintenance service ecosystem for appliances, which is currently absent in rural Rwanda, can sustain a stronger consumer base.	• Increasing the reliance on off-grid solutions in rural areas through mixture of fuel at households can be a key to sustaining green growth and developing collective consumption identities for green growth. • Fig. 10 also showed higher uptake of power stabilizers. It indicated power quality and reliability issues which must be addressed for improving energy affordability and accessibility across the Ubudehe categories. Off-grid transition must address it to sustain disruption from below in rural Rwanda.

## 6 Conclusion and policy implication

This study set the scope for disruption innovation in a bottom-up manner for socially inclusive renewable energy transition for fostering green growth in rural Rwanda. It used a nationally representative dataset of almost 15,000 households and investigated appliance diffusion pattern using social shaping of technology analysis and a binary logistic regression of appliance uptake with socio-economic variables. The pathways for disruptive innovation were envisaged across the lines of micro-entrepreneurship that can support a consumptive-productive-service oriented appliance uptake ecosystem for developing identities of collective consumption behaviour in rural and resource-constrained areas. Such identities of consumption are fundamental for green growth and off-grid renewables planning to ensure power stability and reliability at the household level.

The key conclusions that can be drawn from this study can be broadly divided into three parts. First, the current appliance uptake pattern in rural Rwanda is strongly driven by information and communication technology (ICT) devices like mobile phones and radios. Leveraging this wave of ICT-driven technology diffusion is critical for the development goals of Rwanda and ensuring green growth in Rwanda. This ICT-driven appliance uptake pattern is also critical in ensuring the sustainability of off-grid renewable technologies at the grassroots level through disruptive innovation, as discussed in Table 7.

Secondly, the growing pattern for the uptake of the cooker in Rwanda also presents ae scope for cleaner fuel switching in rural areas. It can be done by replacing the existing cooking fuel mix mainly comprising of firewood with cleaner renewable-based cooking fuel mix. It further creates a scope for disruptive innovation at the community level through off-grid cooking fuel solutions like biomass and solar energy. If the policymakers can converge such initiatives with the broader goal of off-grid electrification, it could significantly impact the inclusive renewable energy transition and foster green growth. The Vision 2020 Umurenge Program (VUP) already provides a platform for such initiatives that should be further leveraged for supporting green growth at a local level in Rwanda.

Thirdly, the social shaping of technology analysis shows that new consumer classes will be created due to the accelerated diffusion of ICT devices. These consumer classes must be made resilient by creating policy and institutional mechanism to develop a collective identity of energy consumption that demands modern and cleaner energy services. It will not only remove the barriers to green growth in low-income settings but also channelise the pathways for efficient delivery of the United Nation’s Sustainable Development Goal – 7 (clean and affordable energy for all).

Developing the future of good energy policymaking through green growth and disruptive innovation at the grassroots level is critical as millions of people will be lifted from extreme poverty within the next decade through the VUP in Rwanda. The findings from this study will aid policymakers and renewable-based enterprises at the grassroots to shape their policies for catering the current wave of household technology diffusion while reducing the uncertainties from off-grid energy systems.

A limitation of this study is in the static use of the concepts of green growth and disruptive innovation at the household-level energy service demand analysis. It was adopted as the scope of this paper was to evaluate the non-income drivers of appliance ownership. The future work should include the income variables in assessing the appliance uptake and social shaping of technology pattern, which can aid in determining the economic utility associated with a specific technology adoption at the household level. It can further derive more in-depth insights into the welfare benefits associated with appliance ownerships and socially inclusive renewable transition.

## CRediT authorship contribution statement

**Olivia Muza:** Conceptualization, Methodology, Writing - original draft, Writing - review & editing. **Ramit Debnath:** Conceptualization, Methodology, Software, Investigation, Data curation, Formal analysis, Visualization, Funding acquisition, Writing - original draft, Writing - review & editing.

## Declaration of competing interest

The authors declare that they have no known competing financial interests or personal relationships that could have appeared to influence the work reported in this paper.

## Footnotes

1 These studies are based on German-context, but the policy implications are widely applied across the Global South context.
