# Supplementary material for: Disruptive innovation for inclusive renewable policy in sub-Saharan Africa: A social shaping of technology analysis of appliance uptake in Rwanda
Source: Renew Energy. 2021 May;168:896–912. doi: 10.1016/j.renene.2020.12.091 (PMC7893685; doi:10.1016/j.renene.2020.12.091)
Supplement: Multimedia component 1 [file mmc1.docx]

**Appendix:**

**Summary statistics**

|  | N | Mean | Std. Deviation | Variance | Kurtosis | |
| --- | --- | --- | --- | --- | --- | --- |
|  | Statistic | Statistic | Statistic | Statistic | Statistic | Std. Error |
| Urban | 14580 | .17 | .378 | .143 | .987 | .041 |
| Rural | 14580 | .83 | .379 | .144 | .969 | .041 |
| Non_poor | 14580 | .67 | .471 | .222 | -1.492 | .041 |
| Moderate_poor | 14580 | .20 | .401 | .161 | .228 | .041 |
| Severe_Poor | 14580 | .13 | .337 | .114 | 2.805 | .041 |
| Q1 | 14580 | .17 | .373 | .139 | 1.208 | .041 |
| Q2 | 14580 | .18 | .386 | .149 | .713 | .041 |
| Q3 | 14580 | .20 | .397 | .158 | .331 | .041 |
| Q4 | 14580 | .21 | .408 | .167 | .006 | .041 |
| Q5 | 14580 | .24 | .429 | .184 | -.566 | .041 |
| U1 | 14580 | .16 | .364 | .133 | 1.543 | .041 |
| U2 | 14580 | .34 | .475 | .225 | -1.562 | .041 |
| U3 | 14580 | .42 | .494 | .244 | -1.901 | .041 |
| U4 | 14580 | .00 | .045 | .002 | 481.167 | .041 |
| Male | 14580 | .74 | .436 | .190 | -.742 | .041 |
| Female | 14580 | .26 | .436 | .190 | -.742 | .041 |
| Radio | 14580 | .46 | .498 | .248 | -1.969 | .041 |
| Mobile | 14580 | .66 | .475 | .225 | -1.558 | .041 |
| TV | 14580 | .10 | .297 | .088 | 5.363 | .041 |
| Sattellite TV | 14580 | .01 | .120 | .014 | 63.811 | .041 |
| DVD Player | 14580 | .06 | .243 | .059 | 10.988 | .041 |
| Decoder | 14580 | .07 | .253 | .064 | 9.657 | .041 |
| Music | 14580 | .01 | .071 | .005 | 192.098 | .041 |
| Computer | 14580 | .03 | .176 | .031 | 26.130 | .041 |
| Cooker | 14580 | .17 | .373 | .139 | 1.187 | .041 |
| Laundry Machine | 14580 | .13 | .333 | .111 | 3.028 | .041 |
| Fan | 14580 | .10 | .299 | .089 | 5.195 | .041 |
| Sewing Machine | 14580 | .03 | .171 | .029 | 28.405 | .041 |
| Refrigerator | 14580 | .00 | .027 | .001 | 1320.909 | .041 |
| Electric generator | 14580 | .00 | .045 | .002 | 481.167 | .041 |
| Hotplate | 14580 | .01 | .113 | .013 | 72.181 | .041 |
| Power stabilizer | 14580 | .02 | .128 | .016 | 55.036 | .041 |
| Camera | 14580 | .00 | .022 | .000 | 2078.571 | .041 |
| Video camera | 14580 | .01 | .071 | .005 | 192.098 | .041 |
| Printer | 14580 | .02 | .130 | .017 | 52.893 | .041 |
| Water Filter | 14580 | .01 | .075 | .006 | 170.727 | .041 |


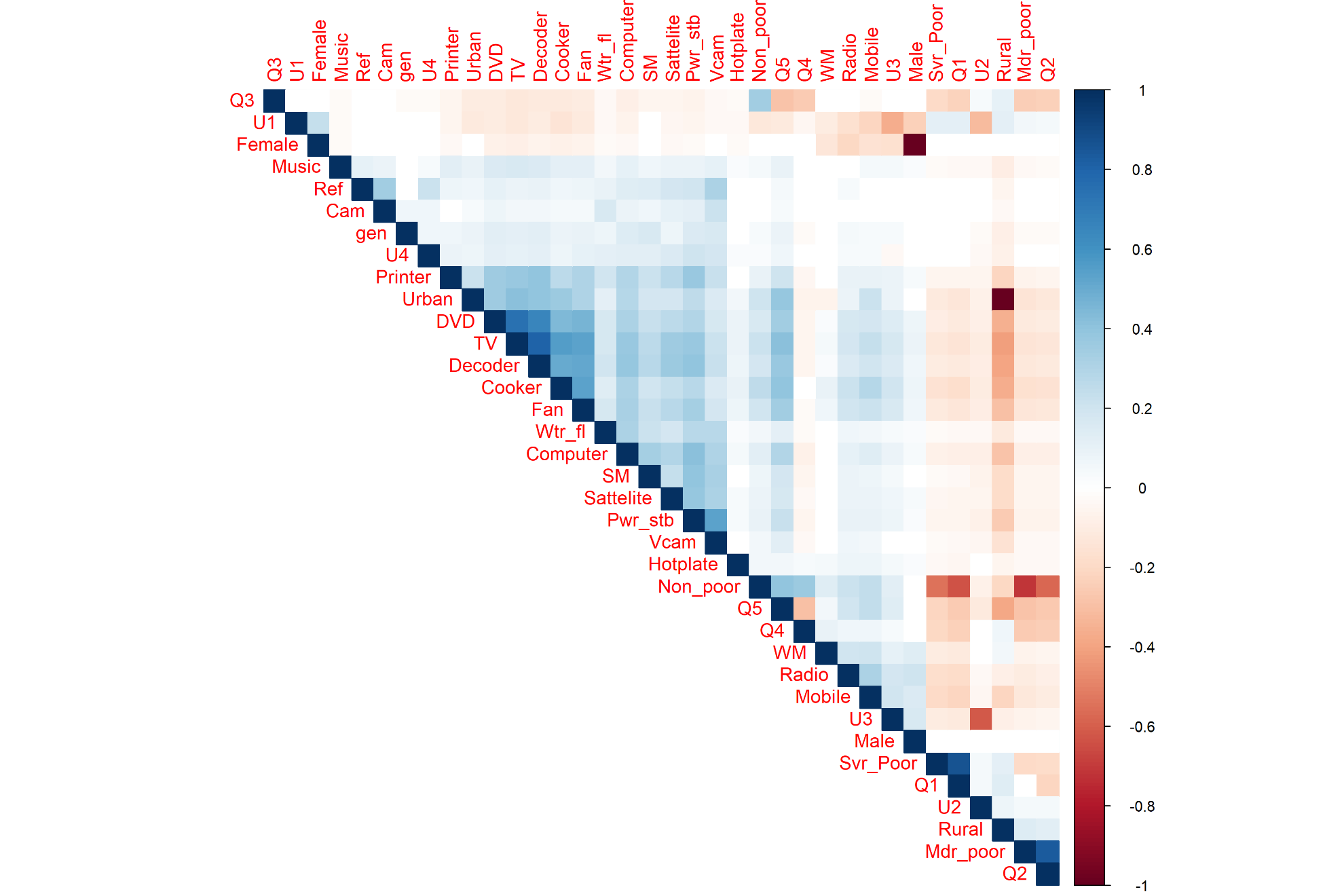


Figure 1. Correlogram of the variables (n =14580).

{Note: Correlations with p-value > 0.01 are considered as insignificant. These values are left blank. Positive correlations are displayed in blue and negative correlations in red colour. Colour intensity is proportional to the correlation coefficients. In the right side of the correlogram, the legend colour shows the correlation coefficients and the corresponding colours}
